# Nationwide Transition From Percutaneous Nephrolithotomy to Endoscopic Combined Intrarenal Surgery in Japan: A Multicenter Survey of Trends and Complications

**DOI:** 10.1111/iju.70510

**Published:** 2026-05-15

**Authors:** Shuzo Hamamoto, Junichi Matsuzaki, Noritaka Ishito, Hiroshi Yokoyama, Hideyasu Iwamoto, Takaaki Inoue, Shinsuke Okada, Toshiyuki Nakamura, Hiroaki Ikoma, Takahiro Yanase, Masahiko Isogai, Rei Unno, Kazumi Taguchi, Takahiro Yasui

**Affiliations:** ^1^ Department of Nephro‐Urology Nagoya City University Graduate School of Medical Sciences Nagoya Japan; ^2^ Department of Urology Oguchi‐Higashi Hospital Yokohama Japan; ^3^ Department of Urology Kurashiki Medical Center Kurashiki Japan; ^4^ Department of Urology Meirikai Yamato Hospital Itabashi Japan; ^5^ Department of Urology Nozaki Higashi Hospital Miyazaki Japan; ^6^ Department of Urology Hara Genitourinary Hospital Kobe Japan; ^7^ Department of Urology Gyotoku General Hospital Ichikawa Japan; ^8^ Department of Urology Tatebayashi Kosei General Hospital Tatebayashi Japan; ^9^ Department of Urology University of Alabama at Birmingham Birmingham Alabama USA

**Keywords:** complication, endoscopic combined intrarenal surgery, mini‐tract, nationwide survey, percutaneous nephrolithotomy

## Abstract

**Objectives:**

To clarify the national transition from conventional percutaneous nephrolithotomy (PCNL) to endoscopic combined intrarenal surgery (ECIRS) and evaluate their safety profiles based on real‐world data.

**Methods:**

A retrospective nationwide survey was conducted at 233 institutions in Japan that provided data on percutaneous kidney stone surgeries performed between April 2019 and March 2022. Annual procedure volumes, surgical positions, tract size, irrigation methods, and perioperative complications were analyzed.

**Results:**

Among 5555 cases, 1618 were PCNL and 3937 were ECIRS. PCNL decreased significantly over the study period, whereas ECIRS increased and accounted for approximately 75% of the cases by 2021. The GMSV position was used in 81% of ECIRS cases, whereas 76% of PCNL cases were performed in the prone position (*p* < 0.001). Mini‐tracts (< 23 Fr) were used in 75.3% of ECIRS and 49.5% of PCNL (*p* < 0.001). Severe bleeding (4.7% vs. 1.5%, *p* < 0.001) and blood transfusion (2.0% vs. 0.7%, *p* = 0.001) occurred more frequently in PCNL than in ECIRS. Postoperative fever (17.1 vs. 19.2%) or sepsis (2.1 vs. 1.5%) were not significantly different.

**Conclusions:**

Although this study was not designed to establish superiority between the procedures, it is the first one to compare the use of PCNL and ECIRS in Japan, and it suggests a marked paradigm shift toward ECIRS. The increasing use of mini‐tracts and the hybrid retrograde‐antegrade approach may be associated with a lower observed rate of bleeding complications. However, postoperative infections remain common with both techniques, highlighting the importance of intrarenal pressure control, optimal patient selection, and meticulous perioperative management.

## 1 Introduction

Urolithiasis is a burdensome urinary tract condition with a prevalence ranging within 1%–20% [1]. Approximately 50% of patients experience recurrence, whereas 10% have multiple stone episodes [2]. In Japan, the incidence of upper urinary tract stones has steadily increased in recent decades, driven by dietary westernization, an aging population, and increasing rates of obesity and metabolic syndrome [3, 4]. This trend has placed a growing burden on urological services and has emphasized the need for safe and effective surgical interventions.

Interventions for urolithiasis have changed dramatically over time. The number of ureteroscopic lithotripsy cases has increased despite a decrease in the number of shock wave lithotripsy cases [5]. The number of percutaneous nephrolithotomies (PCNL) has also increased [5]. Since the first report by Fernström in 1976 [6], PCNL has evolved via various technological innovations, including the development of mini‐tracts [7], endoscopic combined intrarenal surgery (ECIRS) [8], robot‐assisted renal puncture [9], and suction sheaths [10].

A recent meta‐analysis reported that ECIRS achieves a high stone‐free (SF) rate using a hybrid approach that combines retrograde intrarenal surgery (RIRS) with PCNL [11]. Moreover, ECIRS frequently uses miniaturized percutaneous tracts, which reduces the risk of hemorrhage [8]. Despite these advantages, ECIRS has unique limitations: (1) Bilateral irrigation may increase the risk of postoperative fever and sepsis due to elevated intrarenal pressure (IRP) [12, 13]; (2) the technical complexity of combined access presents a clinical challenge and raises concerns regarding widespread adoption of the procedure.

Although ECIRS has gained acceptance in several countries, comprehensive national data on its clinical application and safety profile and comparison with conventional PCNL remain inadequate. Therefore, to clarify the current paradigm shift in kidney stone management, we conducted a nationwide survey on percutaneous endoscopic surgery and analyzed the prevalence of PCNL and ECIRS, as well as the complications associated with both procedures.

## 2 Methods

### 2.1 Study Design

We retrospectively collected data on the numbers of PCNL and ECIRS procedures performed and their intra‐ and postoperative complications from the records of multiple Japanese centers between April 2019 and March 2022. The study was conducted as a nationwide survey with the collaboration of the Japanese Society of Endourology and Robotics. Approval was obtained from the Internal Review Board (IRB) of Nagoya City University Hospital, and the study adhered to the guidelines of the Declaration of Helsinki (IRB number: 60‐21‐0091). Patient consent was obtained in the form of an opt‐out at each institution.

### 2.2 Data Collection

Relevant data were obtained from the medical records of each participating institution. The number of incidents of the following intra‐ and postoperative complications observed during the 3‐year period was collected: ureteral injury, postoperative ureteral stricture, renal pelvic perforation, blood transfusion, bleeding requiring arterial embolisation, fever > 38°C, septic shock, thorax injury, surrounding organ injury, severe complications requiring nephrectomy, and mortality. Complications were defined at the discretion of the surgeon. A survey was conducted on the surgical methods used at each institution, including surgical position, renal puncture methods, tract size, and lithotripsy devices used for PCNL. We collected detailed data on cases with complications, including patient and stone characteristics and surgical methods.

### 2.3 Statistical Analysis

All data were statistically analyzed using EZR for R (version 4.4.1; The R Foundation for Statistical Computing, Vienna, Austria). Nominal variables are expressed as frequencies (%), and Fisher's exact test was used to compare them. The annual trend in the number of surgical procedures performed between 2019 and 2021 was evaluated using Poisson regression. The trend in the annual complication rate was assessed using logistic regression analysis. Regression coefficients were exponentiated to obtain rate ratios or odds ratios with corresponding 95% confidence intervals. Statistical significance was set at *p* < 0.05.

## 3 Results

### 3.1 Treatment Paradigm Shift Against Upper Urinary Tract Calculi

A total of 233 facilities participated in this nationwide survey, and data on 5555 percutaneous cases (1618 PCNL and 3937 ECIRS cases) were collected from 148 facilities over the 3‐year period. A total of 85 institutions (36.5%) did not perform percutaneous surgeries, and 51 institutions (21.9%) had < 10 cases within the 3‐year period. Nine facilities performed > 100 procedures during the 3‐year period.

**FIGURE 1:**
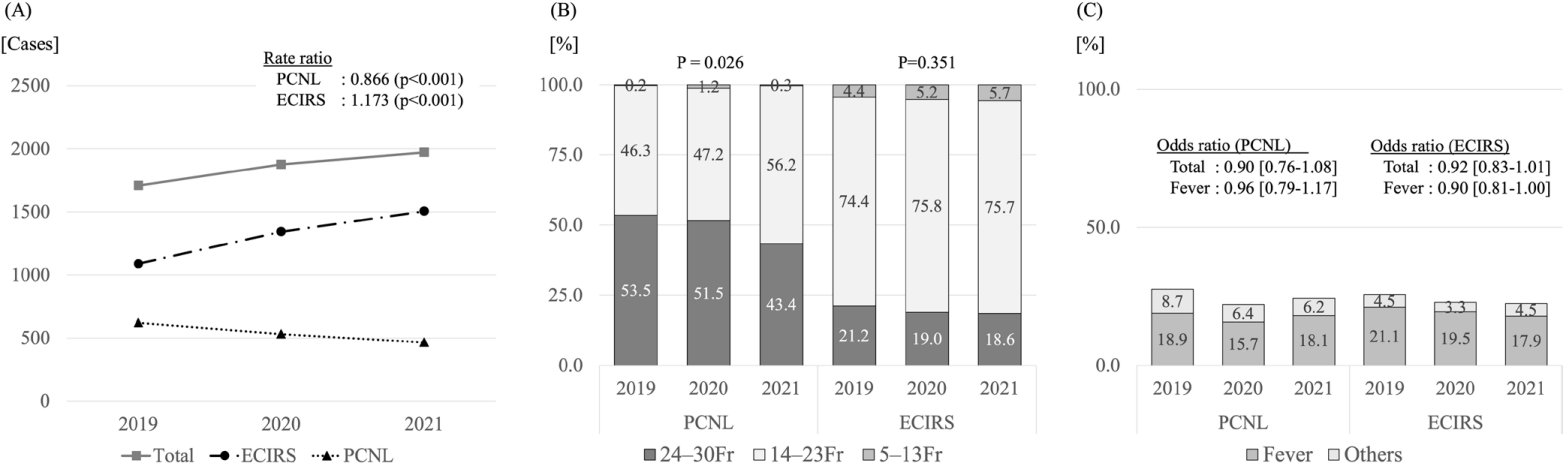
(A) Changes in annual cases of PCNL and ECIRS for the treatment of upper urinary tract calculi. (B) Changes in the proportions of percutaneous tract sizes used in PCNL and ECIRS. (C) Changes in the incidence of complications for percutaneous surgeries.

Changes in the annual numbers of cases of PCNL and ECIRS are shown in Figure 1A. The number of ECIRS procedures gradually increased, whereas the number of PCNL procedures decreased significantly. In 2021, ECIRS accounted for approximately three‐quarters of all percutaneous stone surgeries. Annual changes in the proportions of percutaneous tract sizes used in PCNL and ECIRS are shown in Figure 1B. In PCNL procedures, although the usage rate of conventional‐sized tracts (24–30 Fr) declined, that of mini‐tracts (14–23 Fr) increased significantly (*p* = 0.026). Meanwhile, in ECIRS the trend of the usage rate was similar over time. The total complication rate was 23.2%. No statistically significant change was noted in the annual incidence of complications over time (Figure 1C).

### 3.2 Surgical Differences Between PCNL and ECIRS

Information on the surgical procedures and instruments used was obtained from each institution (Table 1). Surgical positioning differed significantly between the PCNL and ECIRS procedures (*p* < 0.001). Approximately 76% of the PCNL procedures were performed in the prone position. Conversely, 81% of the ECIRS were performed in the oblique position (the Galdakao‐modified Valdivia supine (GMSV) position). Significant differences were observed in the medical instruments used to perform PCNL and ECIRS. In approximately half of the cases of PCNL, conventional tracts with sizes of 24–30 Fr were used, whereas mini‐PCNL tracts were used in 75.3% of ECIRS cases. Gravity irrigation from the percutaneous side was mainly used in PCNL. However, automated irrigation use was higher in ECIRS than in PCNL. Regarding lithotripsy devices, a laser was mainly used in 53.6% of ECIRS cases, whereas in PCNL, a pneumatic device was used for stone fragmentation in addition to a laser.

**TABLE 1:** Information on the surgical procedures.

	PCNL	ECIRS	*p*
Surgical positioning (%)			< 0.001
Prone	76.1	19.0	
Oblique	23.9	81.0	
Percutaneous tract size (%)			< 0.001
24–30 Fr	49.9	19.6	
14–23 Fr	49.5	75.3	
5–13 Fr	0.6	5.1	
Irrigation method from PCNL side (%)			< 0.001
Gravity‐assisted	90.3	74.1	
Manual pumping	8.7	10.1	
Automated pumping	0.9	15.8	
Lithotripsy device from PCNL side (%)			< 0.001
Laser	44.1	53.6	
Pneumatic device	44.9	40.5	
Ultrasonic device	11.0	5.9	

Abbreviations: ECIRS, endoscopic combined intrarenal surgery; Fr, French; PCNL, percutaneous nephrolithotomy.

### 3.3 Comparison of Complications in PCNL and ECIRS

The incidence of postoperative complications did not differ significantly between the PCNL and ECIRS groups (Table 2); however, the types of complications differed between the two techniques. For infectious complications, the incidences of postoperative fever and sepsis in PCNL and ECIRS were 17.1% versus 19.2% and 2.1% versus 1.5%, respectively. However, these differences were not statistically significant. The incidences of severe bleeding and transfusion were significantly higher in PCNL than in ECIRS (4.7% versus 1.5%; *p* < 0.001 and 2.0 versus 0.7; *p* = 0.001). No significant differences were observed in the frequencies of urinary tract complications. The mortality rate was 0.1% for both procedures.

**TABLE 2:** Incidence of postoperative complications in the PCNL and ECIRS groups.

	PCNL	ECIRS	*p*
Total (%)	23.2	23.2	1.000
Infectious complications			
Fever (%)	17.1	19.2	0.134
Sepsis (%)	2.1	1.5	0.213
Bleeding complications			
Severe bleeding (%)	4.7	1.5	< 0.001
Requiring TAE (%)	0.6	0.3	0.262
Requiring transfusion (%)	2.0	0.7	0.001
Urinary tract complications			
Renal pelvic perforation (%)	1.8	1.5	0.57
Ureteral injury (%)	0.5	0.9	0.237
Postoperative ureteral stricture (%)	0.7	0.5	0.475
Other complications			
Thoracic injury (%)	0.2	0.2	1.000
Open conversion (%)	0.2	0.0	0.05
Mortality (%)	0.1	0.1	0.532

Abbreviations: ECIRS, endoscopic combined intrarenal surgery; PCNL, percutaneous nephrolithotomy; TAE, transcatheter arterial embolisation.

## 4 Discussion

ECIRS was initiated in Italy in 2008 [14], and Japan first reported its use in 2011 [8]. Since then, many countries have reported use of the technique. PCNL case numbers have increased gradually worldwide with an increase in the number of urologists performing it [5, 15, 16]; however, no previous reports have compared trends in PCNL and ECIRS. To our knowledge, this is the first nationwide survey to demonstrate trends in percutaneous surgeries for the treatment of upper urinary tract stones and the incidence of their complications in Japan. In total, we analyzed 5555 cases from 233 institutions over a 3‐year period. According to the national database, approximately 3500 percutaneous surgeries are performed annually in Japan [17]. We were able to analyze approximately 50% of the total number of cases performed during the study period. This study demonstrates a significant paradigm shift in the management of large renal and ureteral calculi in Japan, characterized by a transition from conventional PCNL to ECIRS. Over the 3‐year study period, the proportion of ECIRS steadily increased, whereas that of PCNL decreased.

The increasing trend in the use of ECIRS in Japan appears to be driven by several factors: First, with the Japanese medical insurance reforms in 2020, medical remuneration points increased by a factor of 1.5 times the previous amount. ECIRS involves hybrid therapy with RIRS and PCNL, resulting in additional costs for surgeons and medical devices, including access sheaths, lasers, and perfusion fluids. Increased insurance has made this procedure more feasible from a financial perspective. Second, ECIRS has a high SF rate and a low bleeding complication rate. Meta‐analyses have revealed that ECIRS has a 2.52–5.14 times higher SF rate than other percutaneous surgeries [11, 18, 19]. Efficient stone fragmentation using simultaneous antegrade‐retrograde lithotripsy, use of the washout mechanism with retrograde irrigation, and use of the “pass the ball” technique against inaccessible stones using a flexible ureteroscope could help achieve single‐step SF [20]. Furthermore, ECIRS reportedly has fewer complications [8, 11, 18, 19]. In this study, mini‐PCNL tracts were more commonly used in ECIRS than in PCNL, leading to a significantly lower risk of severe bleeding and need for blood transfusion in the ECIRS group. The high SF rate, a key advantage of ECIRS, combined with its low complication rate, may contribute to the increasing adoption of ECIRS in clinical practice. In this study, laser was the preferred lithotripsy technique used in ECIRS, whereas in PCNL, pneumatic devices were used in addition to laser. The technique has shifted from the use of a pneumatic device to bombard a large stone burden, followed by removal with forceps, to the use of a laser to fragment the stone, followed by extraction using retrograde irrigation.

The third factor contributing to the increased trend in ECIRS in Japan might be the shorter learning curve for ECIRS than for PCNL. Previous reports have documented that surgical competence for PCNL can be reached after 45–60 cases [21, 22]. In contrast, ECIRS training requires a learning curve of 16–20 cases to achieve acceptable surgical outcomes [23]. The surgical learning curve is typically shorter for less complex procedures [24]. Although ECIRS may seem more complex than conventional PCNL, ureteroscopy (URS) can simplify the procedure. The most critical steps in ECIRS are renal puncture and tract dilation. ECIRS can confirm renal papillary puncture, contributing to a low risk of renal vascular injury and safe dilation under direct endoscopic vision. Furthermore, the development of the GMSV position, which involves the contralateral leg being fixed and flexed and the ipsilateral leg being slightly extended in the Valdivia supine position, could facilitate more efficient URS without repositioning of the patient [25]. The major surgical position used in ECIRS in this study was the GMSV position, whereas that in PCNL was the prone position. To further examine whether this shift was influenced by changes in case selection, we analyzed a subset of 1874 cases with detailed clinical data (Tables S1 and S2). No significant temporal changes were noted in age, stone size, or CT attenuation values, and ECIRS cases tended to involve comparable or slightly more complex stones than PCNL cases across all years. These findings suggest that the increased use of ECIRS cannot be explained solely by changes in case selection.

We found that the annual number of percutaneous surgeries performed to treat upper urinary tract stones in Japan has gradually increased. The annual incidence of intra‐ and postoperative complications did not differ significantly between years. Febrile complications are one of the most concerning issues after percutaneous surgeries. The relatively high incidence of postoperative fever observed in both groups highlights that infectious complications remain a significant clinical challenge. The incidences of postoperative fever and sepsis after ECIRS in this study were 19.2% and 1.5%, respectively, which were similar to those observed after PCNL (17.1% and 2.1%, respectively). These results were also similar to those reported in previous meta‐analyses [11, 18, 19] but were much higher than those reported in one previous study (fever, 10.8%; sepsis, 0.5%) [26]. This may reflect the effect of mini‐PCNL tract use or bilateral irrigation on IRP. An elevated IRP during endoluminal surgery can promote pyelovenous backflow and systemic bacterial translocation, resulting in infectious complications. Mini‐PCNL tracts can reduce the risk of intraoperative bleeding [8]; however, there are concerns about increasing IRP. Wu et al. reported that the average IRP during mini‐PCNL was approximately two times higher than that during conventional PCNL, resulting in a significantly higher risk of postoperative fever [27]. Bilateral irrigation may also cause an increase in IRP during ECIRS. We recently reported that automated irrigation and pressure management resulted in significantly fewer febrile events in ECIRS than when gravity‐based manual irrigation was used, highlighting the importance of meticulous intraoperative techniques [13].

Ureteral complications remain a concern in PCNL and ECIRS. Our survey identified cases of ureteral injury, perforation, and postoperative stricture, which may have been related to the manipulation of stone fragments or difficult scope retrieval. The incidence of ureteral injury around the ureteropelvic junction is significantly higher in the prone position than in the GMSV position during ECIRS [28]. In the GMSV position, it runs upward, whereas in the prone position, the ureter runs downward from the renal pelvis, and the stone fragment can easily migrate into the ureter from the renal pelvis, contributing to ureteral complications [28]. These findings underscore the need for careful ureteral access and the consideration of preoperative stenting in high‐risk patients.

This study had some methodological limitations. First, as the data in this study were collected only from participating institutions, they cannot be considered a reflection of all registry results. However, this survey provides valuable epidemiologic insight, covering approximately half of all PCNLs performed nationally. As such, it complements existing registry data from the CROES [29] and BAUS databases [30], expanding global understanding of percutaneous stone surgery trends in diverse healthcare systems. Second, the definitions of the complications were not standardized, and they were defined and reported at the discretion of each surgeon. Complication severity (e.g., Clavien–Dindo grade) and patient‐level characteristics such as stone size or Guy's stone score were unavailable. These items might have introduced selection and reporting bias, which limits the reliability and comparability of the reported complication rates. Furthermore, no detailed data on infectious factors, such as urine culture, antibiotic use, or preoperative stent placement, were available, and the reason for the high infection rate in this study is unknown. Third, operative time, reintervention rate, and cost data were not captured. Finally, the choice between PCNL and ECIRS was based on surgeon preference and facility preparation. ECIRS is performed at relatively high‐volume centers. This lack of randomization might have caused selection bias, even though no significant differences were observed in the patient demographics. Despite these limitations, the survey encompassed approximately half of all percutaneous renal surgeries performed in Japan, providing the largest national overview to date and reflecting real‐world practice across a broad range of centers.

In conclusion, ECIRS has emerged as the predominant percutaneous stone surgery in Japan, likely driven by both clinical and socio‐economic factors, including a lower observed rate of bleeding complications compared with PCNL, a comparable incidence of infectious complications, and changes in healthcare policy and reimbursement structures. However, these findings should be interpreted cautiously due to the retrospective design and lack of standardized data.

## Author Contributions

**Noritaka Ishito:** investigation, writing – review and editing. **Takaaki Inoue:** conceptualization, investigation, writing – review and editing. **Shinsuke Okada:** conceptualization, investigation, writing – review and editing. **Toshiyuki Nakamura:** investigation. **Junichi Matsuzaki:** investigation, writing – review and editing. **Hideyasu Iwamoto:** investigation, writing – review and editing. **Hiroshi Yokoyama:** investigation, writing – review and editing. **Shuzo Hamamoto:** conceptualization, methodology, data curation, investigation, writing – review and editing, writing – original draft, project administration, formal analysis. **Masahiko Isogai:** investigation. **Takahiro Yasui:** supervision. **Takahiro Yanase:** writing – review and editing. **Rei Unno:** writing – review and editing. **Hiroaki Ikoma:** formal analysis. **Kazumi Taguchi:** supervision.

## Ethics Statement

The protocol for this research project has been approved by a suitably constituted Ethics Committee of the institution and it conforms to the provisions of the Declaration of Helsinki. Committee of Nagoya City University Hospital, Approval No. 60‐21‐0091.

## Consent

Patient consent was obtained in the form of an opt‐out at each institution.

## Conflicts of Interest

Takahiro Yasui is an Editorial Board member of International Journal of Urology and a co‐author of this article. To minimize bias, he was excluded from all editorial decision‐making related to the acceptance of this article for publication.

## Supporting information


**Table S1:** Temporal changes in patient and stone characteristics among the analyzed cases (*n* = 1874).
**Table S2:** Comparison of patient and stone characteristics between PCNL and ECIRS within each study year.

## Data Availability

The datasets generated or analyzed in the present study are available from the corresponding author upon reasonable request.
