# Supplementary material for: Nationwide Transition From Percutaneous Nephrolithotomy to Endoscopic Combined Intrarenal Surgery in Japan: A Multicenter Survey of Trends and Complications
Source: Int J Urol. 2026 May 15;33:e70510. doi: 10.1111/iju.70510 (PMC13179465; doi:10.1111/iju.70510)
Supplement: Supplementary file 1 — Table S1: Temporal changes in patient and stone characteristics among the analyzed cases (n = 1874). Table S2: Comparison of patient and stone characteristics between PCNL and ECIRS within each study year. [file IJU-33-0-s001.docx]

Supplementary Table 1: Temporal changes in patient and stone characteristics among the analyzed cases (n=1,874)

|  | 2019 | 2020 | 2021 | p-value |
| --- | --- | --- | --- | --- |
| age (years), median [IQR] | 61 [52, 71] | 62 [52, 71] | 62 [52, 71] | 0.91 |
| sex (male), n (%) | 335 (55.8) | 364 (60.7) | 424 (63.7) | 0.028 |
| stone size (mm), median [IQR] | 35.0 [25, 50] | 33.0 [23, 48] | 34.0 [25, 50] | 0.42 |
| CT attenuation, median [IQR] | 1160 [840, 1380] | 1180 [890, 1420] | 1165 [880, 1420] | 0.37 |
| Staghorn, n (%) | 337 (55.4) | 281 (46.8) | 323 (48.5) | 0.021 |

Supplementary Table 2: Comparison of patient and stone characteristics between PCNL and ECIRS within each study year

|  | 2019 | | | 2020 | | | 2021 | | |
| --- | --- | --- | --- | --- | --- | --- | --- | --- | --- |
|  | PCNL (n=241) | ECIRS (n=367) | p-value | PCNL (n=202) | ECIRS (n=398) | p-value | PCNL (n=197) | ECIRS (n=469) | p-value |
| age (years), median [IQR] | 64 [50, 72] | 60 [53, 70] | 0.314 | 62 [52, 72] | 62 [52, 71] | 0.849 | 62 [52, 71] | 62 [52, 72] | 0.855 |
| sex (male), n (%) | 123 (51.0) | 212 (57.8) | 0.122 | 123 (60.9) | 241 (60.6) | 1 | 123 (62.4) | 301 (64.2) | 0.735 |
| stone size (mm), median [IQR] | 33.9 [24.6, 46.0] | 36.25 [26.0, 52.0] | 0.04 | 31.0 [21.2, 41.5] | 34.0 [24.0, 50.0] | 0.025 | 32.0 [24.0, 45.0] | 35.0 [25.0, 50.0] | 0.106 |
| CT attenuation, median [IQR] | 1131 [780.8. 1335.5] | 1180 [864.0,1415.0] | 0.044 | 1141 850, 1367.5] | 1196 [899, 1440] | 0.085 | 1145 [829, 1400] | 1162 [879.9, 1423.3] | 0.597 |
| Staghorn, n (%) | 116 (48.1) | 221 (60.2) | 0.004 | 70 (34.7) | 211 (53.0) | < 0.001 | 79 (40.1) | 244 (52.0) | 0.006 |
